# Mechanisms of Xenogeneic Baboon Platelet Aggregation and Phagocytosis by Porcine Liver Sinusoidal Endothelial Cells

**DOI:** 10.1371/journal.pone.0047273

**Published:** 2012-10-30

**Authors:** Qiang Peng, Heidi Yeh, Lingling Wei, Keiichi Enjyoj, Zurab Machaidze, Eva Csizmad, Christian Schuetz, Kang Mi Lee, Shaoping Deng, Simon C. Robson, James Markmann, Leo Buhler

**Affiliations:** 1 Division of Transplant Surgery, Massachusetts General Hospital, Harvard Medical School, Boston, Massachusetts, United States of America; 2 Department of Gastroenterology, Liver Center, Beth Israel Deaconess Medical Center, Harvard Medical School, Boston, Massachusetts, United States of America; 3 Surgical Research Unit, Department of Surgery, University Hospital Geneva, Geneva, Switzerland; Institut National de la Santé et de la Recherche Médicale, France

## Abstract

**Background:**

Baboons receiving xenogeneic livers from wild type and transgenic pigs survive less than 10 days. One of the major issues is the early development of profound thrombocytopenia that results in fatal hemorrhage. Histological examination of xenotransplanted livers has shown baboon platelet activation, phagocytosis and sequestration within the sinusoids. In order to study the mechanisms of platelet consumption in liver xenotransplantation, we have developed an *in vitro* system to examine the interaction between pig endothelial cells with baboon platelets and to thereby identify molecular mechanisms and therapies.

**Methods:**

Fresh pig hepatocytes, liver sinusoidal and aortic endothelial cells were isolated by collagenase digestion of livers and processing of aortae from GTKO and Gal+ MGH-miniature swine. These primary cell cultures were then tested for the differential ability to induce baboon or pig platelet aggregation. Phagocytosis was evaluated by direct observation of CFSE labeled-platelets, which are incubated with endothelial cells under confocal light microscopy. Aurintricarboxylic acid (GpIb antagonist blocking interactions with von Willebrand factor/vWF), eptifibatide (Gp IIb/IIIa antagonist), and anti-Mac-1 Ab (anti-α_M_β_2_ integrin Ab) were tested for the ability to inhibit phagocytosis.

**Results:**

None of the pig cells induced aggregation or phagocytosis of porcine platelets. However, pig hepatocytes, liver sinusoidal and aortic endothelial cells (GTKO and Gal+) all induced moderate aggregation of baboon platelets. Importantly, pig liver sinusoidal endothelial cells efficiently phagocytosed baboon platelets, while pig aortic endothelial cells and hepatocytes had minimal effects on platelet numbers. Anti-MAC-1 Ab, aurintricarboxylic acid or eptifibatide, significantly decreased baboon platelet phagocytosis by pig liver endothelial cells (*P*<0.01).

**Conclusions:**

Although pig hepatocytes and aortic endothelial cells directly caused aggregation of baboon platelets, only pig liver endothelial cells efficiently phagocytosed baboon platelets. Blocking vWF and integrin adhesion pathways prevented both aggregation and phagocytosis.

## Introduction

An increasing shortage of suitable donor livers has led to high rates of mortality of those patients awaiting liver allotransplantation. Alternatives to deceased or living donor liver transplantation include xenotransplantation, with genetically modified swine being the preferred donor animal [1]. The development of pigs, which lack the alpha1,3-galactosyltransferase gene (GTKO) and express human complement regulatory proteins (hCRP) [2], has largely eliminated hyperacute rejection of pig solid organs transplanted into primates [3]. Heart [4] and kidney xenotransplants [5] can survive for up to 3–6 months. However, baboon recipients of GTKO pig livers survive for less than 10 days, with profound thrombocytopenia accompanied by fatal bleeding being major limitations [6].

The endothelial lining of the liver sinusoids differ from the heart and kidney in lacking a well developed basement membrane. Its organization into a fenestrated monolayer exposes hepatocytes and macrophages (Kupffer cells) directly to the blood circulation, and both cell types have been shown to play a role in platelet homeostasis [7]. However, liver sinusoidal endothelial cells (LSEC) are also unique among endothelial cells in their ability to clear particulate matter from the bloodstream, and many scavenger receptors have been identified on LSEC that facilitate this process [8], whether purely for clearance function or as part of LSEC's antigen presentation function. Interestingly, a recent study showed that human platelets perfused through an *ex vivo* pig liver largely disappear from the circulation within 15 minutes, and can subsequently be found in phagosomes of the LSEC [9].

The receptors involved in platelet phagocytosis by LSEC are not yet well-defined. Receptors which mediate platelet phagocytosis by other cell types may be expressed on LSEC. Indeed, a recent report indicated that the asialoglycoprotein receptor 1 (ASGR1) that is responsible for platelet phagocytosis by Kupffer cells and hepatocytes is also found on porcine liver endothelial cells [10]. Another potential candidate is MAC-1, the ß-2 integrin receptor that mediates platelet phagocytosis by dendritic cells and neutrophils, which is also upregulated on endothelial cells in response to injury and inflammation [11].

To investigate the latter pathways involved in platelet phagocytosis by LSEC, we have established an *in vitro* co-culture system with labeled platelets and hepatocytes or endothelial cells, which allowed us to selectively block putative receptors and examine the effect on platelet aggregation and phagocytosis.

## Materials and Methods

### Cell isolation and culture

Aortic endothelial cells were isolated from 5–10 cm segments of the thoracic aorta from MGH miniature swine (Gal+and GTKO), using a method adapted from that previously described [12]. Briefly, the segment was filled with 0.1% collagenase A (Sigma, St Louis, MO) in PBS for 15 min. Endothelial cells were released by mechanical disruption, then resuspended in culture medium EGM-2 (Lonza, Portsmouth County, NH) containing 10% FBS, penicillin (100 units/ml), streptomycin (100 µg/ml) and amphotericin B (2.5 µg/ml). Any cells that remained floating after 24 hours were removed from the culture flask.

For hepatocyte isolation, the portal vein and hepatic artery of swine were cannulated *in situ* and immediately flushed with ice cold UW solution prior to cardiac arrest and excision of the liver. Initial *ex vivo* perfusion was performed for 15 minutes with calcium-free hepatocyte wash medium (Invitrogen, Carlsbad, CA) at 10 ml/min in a sterile device consisting of a reservoir with an oxygenator and air trap, followed by0.1% collagenase A (Sigma) in PBS for 15 minutes. The liver was then cut into 2–5 mm pieces in hepatocyte wash medium (Invitrogen) and manually passed through a 100 µm nylon mesh. Cells were cultured in hepatocyte culture medium (Lonza).

Donor pigs were housed at the MGH animal facility, receiving free water and food. Liver procurement was performed in the operating room under general anesthesia. Donor pigs were sacrificed under general anesthesia after harvesting of the liver by intravenous overdose of pentobarbital.

For liver endothelial cell isolation, the collagenase perfusate was collected and 10% FBS added to inactivate the enzyme. After centrifugation at 50 g for 10 min, cells in the supernatant were washed with Dulbecco's modified Eagle's medium (DMEM, Invitrogen) containing 10% FBS and seeded in a gelatin-coated T25 cm^2^ flask (Santa Cruz, Santa Cruz, CA) in EGM-2. After 1 hour at 37°C, the non-adherent cells were collected and cultured in gelatin-coated flasks in EGM-2 containing 10% FBS, 2 mmol/L L-glutamine (GIBCO, Billings, MT), 100 µg/mL penicillin/streptomycin and 100 µg/mL endothelial cell growths supplement (Lonza). Cell viability was determined by trypan blue exclusion.

The use of primary cells isolated or cultured from pig and baboon tissues for these experiments was reviewed and approved by the Massachusetts General Hospital Subcommittee on Research Animal Care.

### Immunofluorescence

To confirm cell type and purity, cultured cells were grown in slide chambers (In Vitro Scientific, Pagoda Tree Court, Sunnyvale, CA) for 24 hours, fixed with 2%paraformaldehyde,and stained with mouse anti-pig CD31 (LCI-4, Santa Cruz), and hepatocyte specific factor antibodies (Santa Cruz) at 1∶100 dilution for 1 hour at 4°C. PE-conjugated anti mouse IgG (Jackson immuno research, West Baltimore Pike, West Grove, PA) was added (1∶100 dilution in DPBS) for 1 hour at 4°C. We also tested uptake of acetylated low density lipoprotein, labeled with 1,1\′-dioctadecyl – 3,3,3\′,3\′-tetramethyl-indocarbocyanine perchlorate (DiI-Ac-LDL) (Biomedical Technologies Inc, Stoughton, MA) to confirm the nature of endothelial cells. Briefly, we diluted DiI-Ac-LDL to 10 ug/ml in complete growth media that was added to cells and incubated for 4 hours at 37°C. After washing with PBS, cells were analyzed under confocal microscopy.

### Flow cytometry

Liver sinusoidal endothelial cells were harvested by EDTA solution and spun at 1200 rpm for 5 min. The pellets were fixed by 2% formaldehyde in PBS/pH 7.4 for 15 min. The cells were washed 3 times by PBS and separated as aliquots of 100 µl cell suspension (1×10^6^ cells) in12×75 mm tubes for staining. 100 µl diluted antibody (CD14-FITC and Alexa fluor 647 Conjugated MAC-1, 1∶100) were added. Tubes were kept on ice at 4°C and incubated for 30 minutes in dark. Cells were washed 3 times with FACS buffer, and suspended in 400 microL FACS buffer for analysis.

### Platelet isolation and CFSE labeling

10 ml blood was drawn from baboons or pigs into citrate tubes and centrifuged at 150 g for 15 min at 22°C. The supernatant, consisting of platelet-rich plasma (PRP), was collected and centrifuged at 1500 g for 15 min at 22°C twice to obtain purified platelets (washed platelets) and platelet poor plasma (PPP). Washed platelets were resuspended in 15 ml carboxyfluorescein diacetate succinimidyl ester (CFSE, Sigma, St. Louis, MO) work solution (0.5 µM) for 30 min at 37°C and then 15 ml 10% FBS was added. The platelet solution was then centrifuged at 300 g for 15 min at 22°C. The resulting pellet was washed in 25 ml tyrode solution and platelets counted in a particle counter Z1 (Counter Electronics LTD, UK). Apyrase (0.1 mg/ml) was added to the tyrode solution as anti-aggregant for washed platelets. Samples were diluted to a final concentration of 5×10^8^platelets/ml with PBS for experiments.

This study involving baboons was approved by the Massachusetts General Hospital Subcommittee on Research Animal Care # 2010N000177. Donor baboons were housed at the MGH animal facility, with major efforts to ensure health and welfare, including veterinary care, enriched food and social interactions. Baboons were housed indoor (in cage), cage size 1.5 m×1.5 m×1.5 m, and the environmental condition was light by dark cycle. Baboons received food and water ad libitum. Blood was drawn under general anesthesia(ketamine intramuscular injection, 6 mg/kg).

### Platelet aggregation test

Platelet aggregation was measured using the two-sample, four-channel; model 560 Ca Lumi-aggregometer (Chronolog Corp, Havertown, PA). Platelet-poor plasma was used as a reference sample for setting the baseline optical density (0%). 2×10^6^ liver sinusoidal endothelial cells, hepatocytes or aortic endothelial cells were mixed with 2×10^8^baboon or porcine washed platelets (400 µl) in siliconized glass cuvettes (Chrono Log Corporation). Shear stress was applied by magnet induced agitation of the platelet suspension and percent change in light optical density was measured for 10 minutes [13].

### Platelet phagocytosis

Cultured cells were transferred, at confluence, to a glass bottom plate (D35-10-0-N, in vitro scientific). 5×10^7^ CFSE labeled platelets were added for 1 h at 37°C and the plate was then washed twice with PBS. At end of incubation, 2 ml of 5 µMCellTracker™ Blue CMAC (7-amino-4-chloromethylcoumarin) (C2110, Invitrogen) working solution was added for 30 minutes. Cells were fixed with 2% paraformaldehyde and examined under confocal microscopy (Zeiss LSM 510 Meta Live Cell Confocal Microscope, Thornwood, NY). The number of CFSE-platelets inside cells was counted and phagocytosis index defined as the number of platelets ingested per 100 cells. Data are the average of 3 independent experiments. To test the ability of various drugs to block platelet phagocytosis, liver endothelial cells were pre-cultured for 30 minutes with aurintricarboxylic acid (10 µg/ml, GPIb inhibitor, Sigma), eptifibatide (10 µg/ml, GPIIb/IIIa receptor antagonist, Cor Therapeutics Inc), ICRF44 (10 µg/ml, CD11b monoclonal antibody, Mac-1 antibody, BioLegend, San Diego,CA) prior to the addition of platelets.

### Statistical analysis

Data are shown as mean ±standard deviation. Statistical analysis was conducted using Student's t-test or one way ANOVA. *P*<0.05 was considered to be statistically significant. Statistics were calculated using Prism 4.0 software (GraphPad Software Inc, USA).

## Results

### Isolation of fresh of aortic or liver sinusoidal endothelial cells and hepatocytes

**Figure 1:**
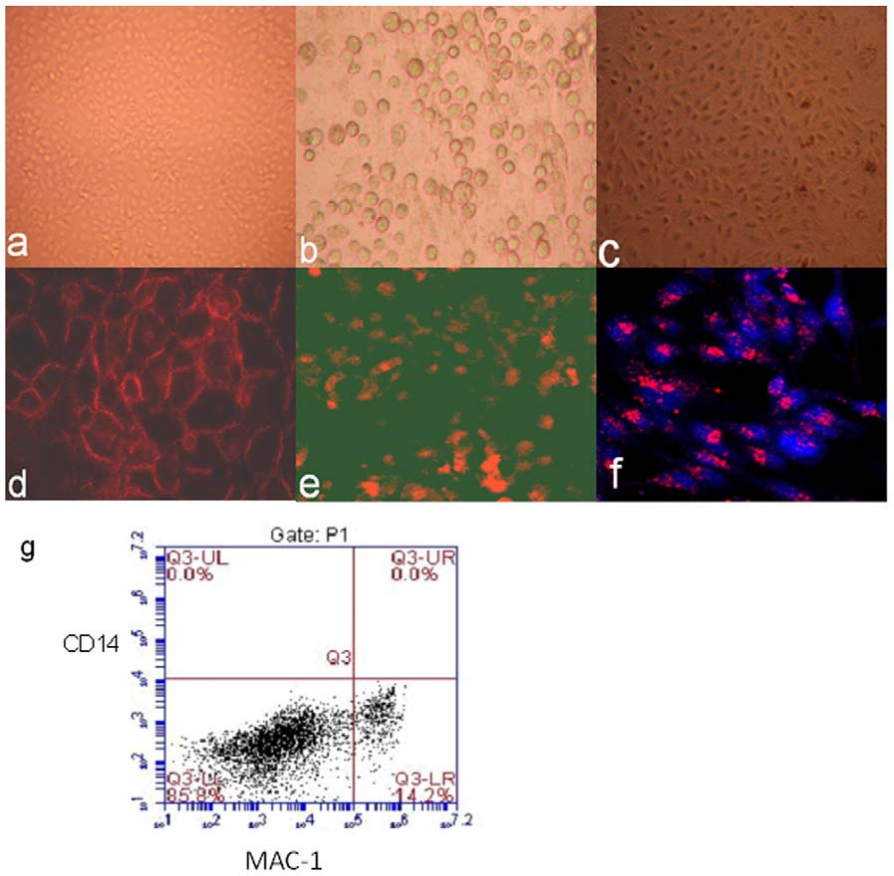
Cell culture and identification. Porcine aortic endothelial cells (a), hepatocytes (b), and liver sinusoidal endothelial cells (c) were in culture. The primary cells where then tested by anti- CD31 antibody(d), anti-hepatocyte specific antigen antibody (OCH1E5) (e), and Dil-Ac-LDL up-take(f) and examined under fluorescence microscope.To understand MAC-1 receptor expressed in liver sinusoidal endothelial cells,flow cytometry test for MAC-1 and CD14 in cells was done(g). The expression rate of Mac-1 in cells was 14.2%. In another hand, CD14 expression in cells was negative. It meaned that all the cells were not Kupffer cells.

Aortic endothelial cells became confluent after 14 days and were CD31+ (Figure 1a and d). Hepatocytes became adherent and were identified after 7 days and were OCH1E5+ (Figure 1b and e).Liver sinusoidal endothelial cells became confluent after 10 days and were identified by Dil-LDL uptake (Figure 1c and f). Flow cytometry showed that liver sinusoidal endothelial cells were positive for MAC-1 up to 14%, whereas negative for CD14 (macrophage marker) (Figure 1 g). The viability of cells used for *in vitro* tests was greater than 90% (data not shown).

### Porcine cells induce aggregation of resting xenogeneic baboon platelets, but not of porcine platelets

**Figure 2:**
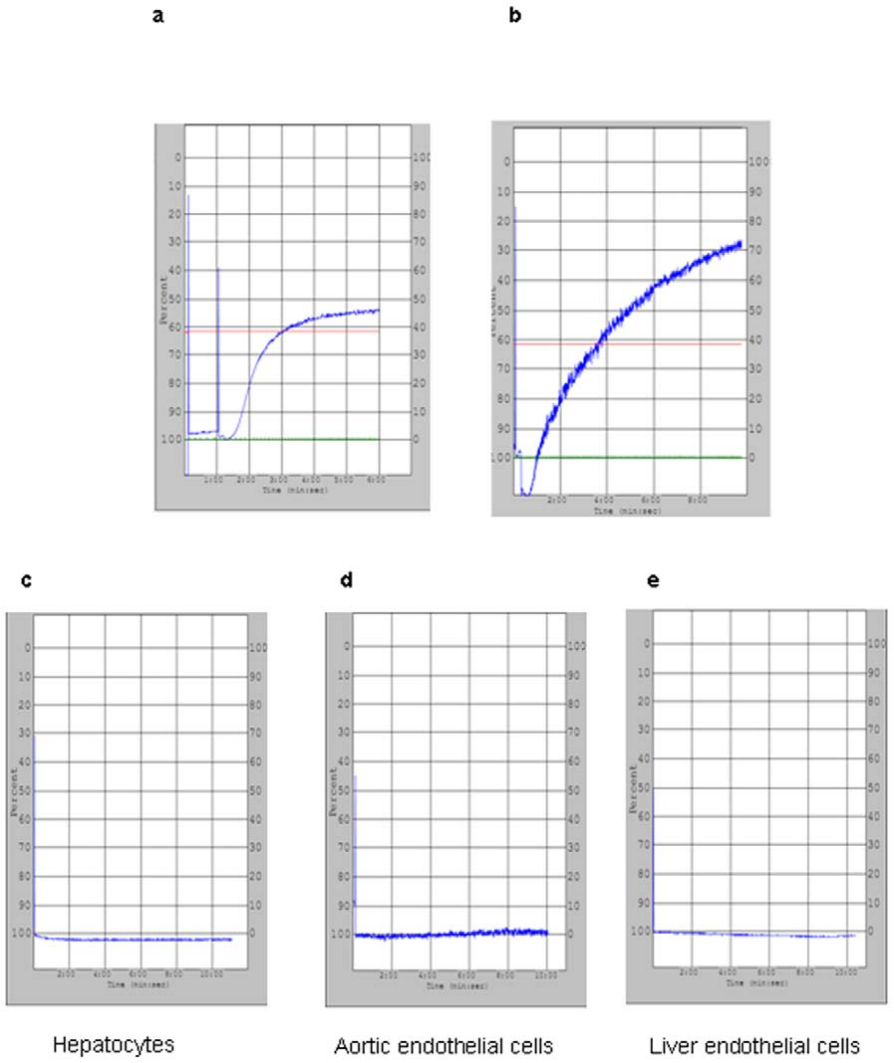
Pig platelets aggregation in different pig cells. (a) Pig or (b) baboon platelets (2×10^8^) were stimulated by collagen (0.5 ug/ml) and showed strong aggregation. When pig platelets (2×10^8^) were mixed with (c) pig hepatocytes, (d) pig aortic endothelial cells and (e) pig liver endothelial cells separately (2×10^6^), no platelet aggregation was observed.

**Figure 3:**
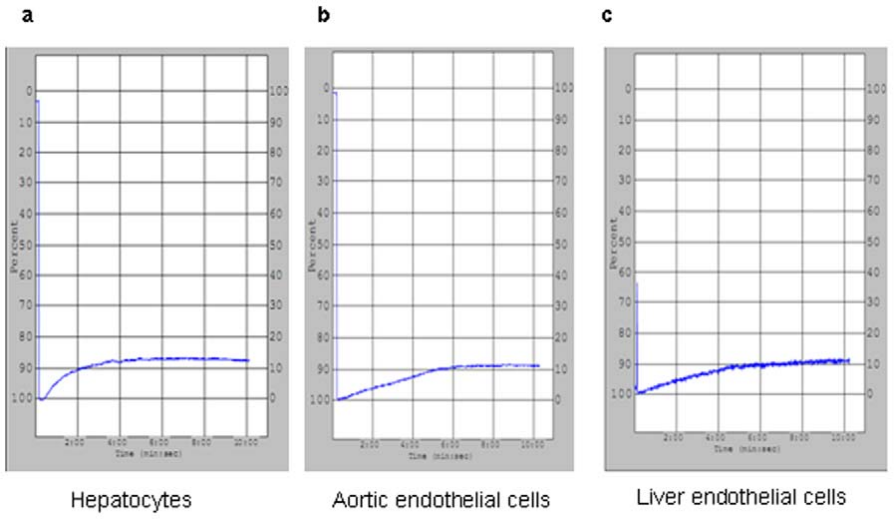
Baboon platelets aggregation in different pig cells. Baboon platelets (2×10^8^) were mixed with (a) pig hepatocytes, (b) pig aortic endothelial cells and (c) pig liver endothelial cells separately (2×10^6^).Mild platelet aggregation was observed, corresponding to 10.8±0.7%, 10.5±0.4%, and 10.4±0.4%. There was no significant difference between these various groups (*P*>0.05).

Since platelet aggregates have been found in porcine livers perfused with human platelets (9), we began by quantifying the clumping response of baboon platelets exposed to porcine cells in culture. Soluble collagen efficiently induced platelet aggregation in both species (Figure 2a and b). While porcine aortic endothelial cells (AEC), liver sinusoidal endothelial cells (LSEC) or hepatocytes did not induce porcine platelet aggregation (Figure 2 c–e), they did induce baboon platelet aggregation with changes in light optical density of 10.8±0.7%, 10.4±0.4%, and 10.5±0.4%, respectively (no statistical difference among these groups, *P*>0.05, Figure 3a–c).

### Pig LSEC preferentially phagocytose resting baboon platelets

**Figure 4:**
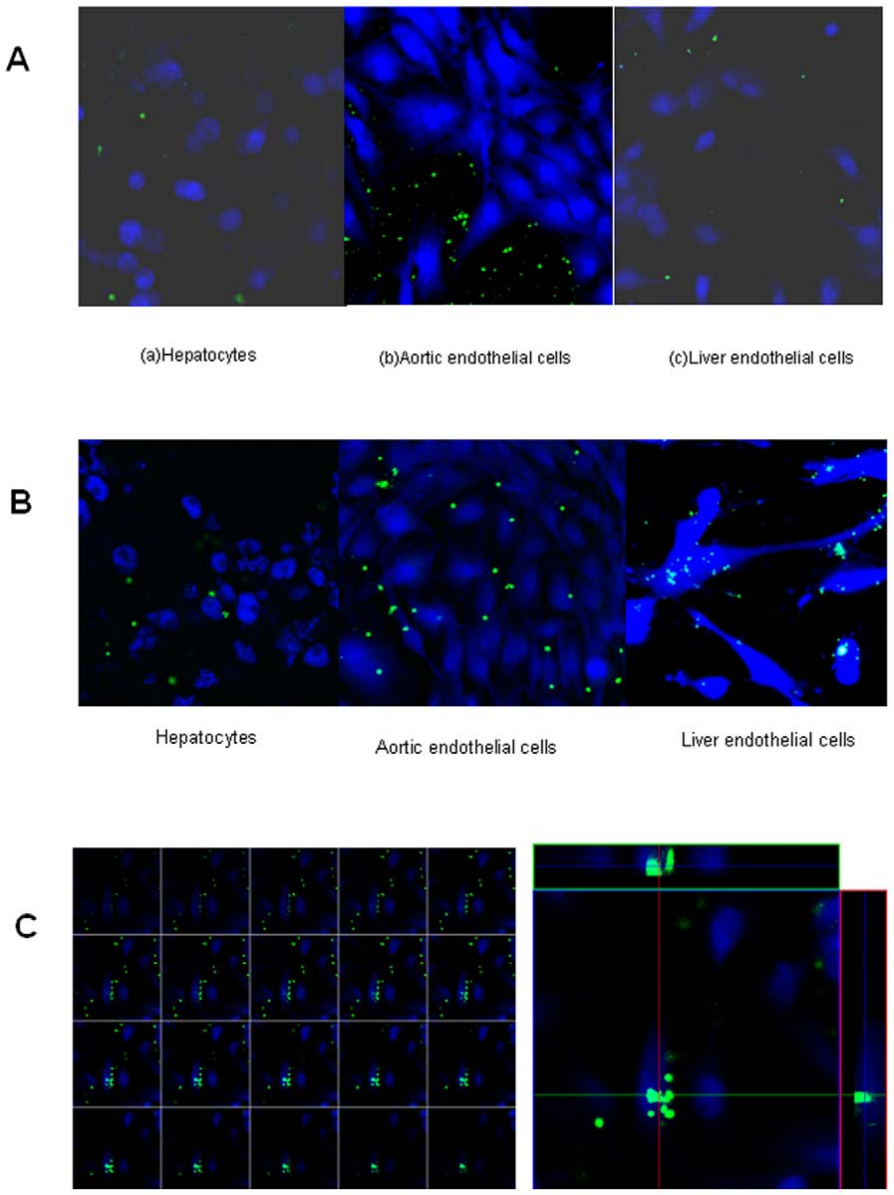
Platelets phagocytosis in different cells. In (A) CFSE labeled porcine platelets (green) were mixed with pig hepatocytes, aortic endothelial cells and liver endothelial cells (stained by CellTracker™ Blue CMAC, blue), co-cultured for 1 h at 37°C. No phagocytosis was observed (confocal microscopy×400).In (B), CFSE labeled baboon platelets (green) were mixed with pig hepatocytes, aortic endothelial cells and liver endothelial cells (stained by CellTracker™ Blue CMAC, blue), co-cultured for 1 h at 37°C. Platelet internalization by hepatocytes, aortic endothelial cells and liver endothelial cells was absent, mild and strong, respectively (confocal microscopy ×400). In (C), baboon platelets (labeled by CFSE, green) are rapidly internalized by pig liver endothelial cells (stained by CellTracker™7-amino-4-chloromethylcoumarin, blue) (confocal microscopy ×400). The left panel shows the sequential section analysis of a single endothelial cell that has internalized platelets, the sections go from top to bottom. The right panel shows the analysis of one section with simultaneous indication of location of platelets within the endothelial cell, indicating that these are clearly internalized and not on the cell surface.

**Figure 5:**
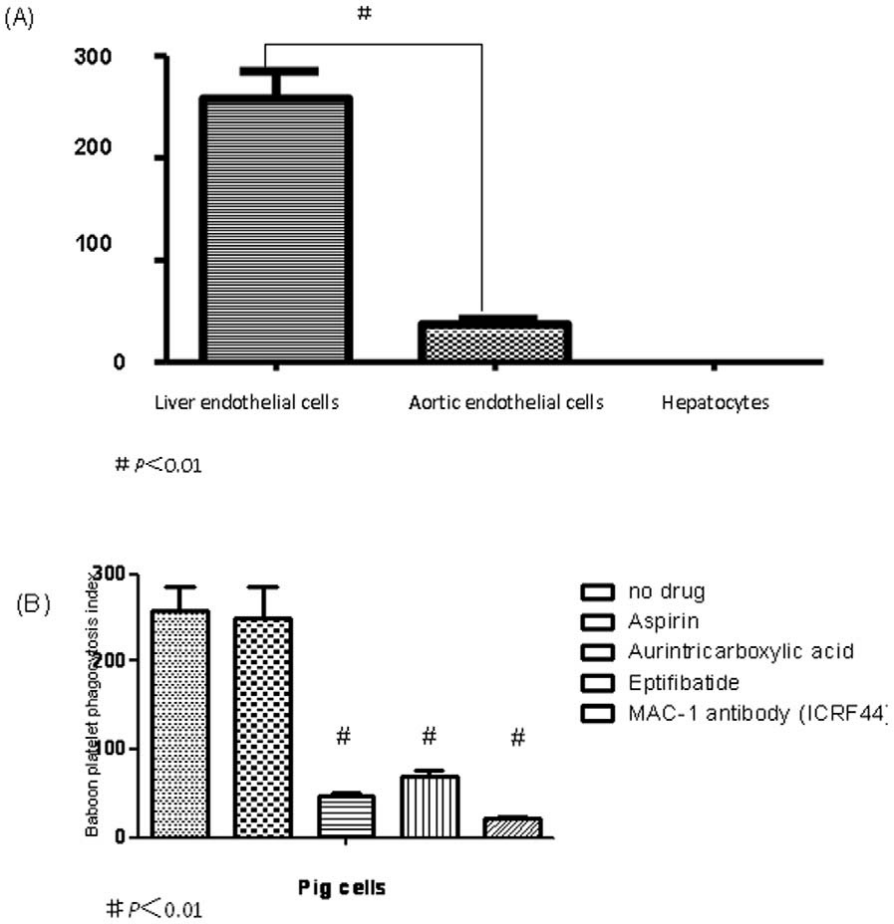
Platelets phagocytosis blocked by different drugs. Phagocytosis index of baboon platelets and pig cells (phagocytosis index = number of platelets internalized per 100 cells and expressed as means ± SD of values from three independent experiments. In (A), no drug was added to the pig cell- baboon platelet combinations (#is statistically significant p<0.01). In (B), various drugs (aspirin, aurintricarboxylic acid, eptifibatide, or ICRF44 were added to the pig liver endothelial cell- baboon platelet combinations, (#is significantly lower when compared with no drug, *P*<0.01).

Previous studies suggest that phagocytosis, as well as aggregation, contribute to the disappearance of primate platelets circulating through porcine livers. We therefore tested the ability of various porcine cell types to phagocytose primate platelets *in vitro*. Porcine AEC, LSEC, and hepatocytes did not phagocytose porcine platelets (Fig. 4A). Baboon platelets were likewise not consumed by porcine hepatocytes (Fig. 4B), and porcine AEC showed minimal ability to internalize baboon platelets, with a phagocytosis index of 37±5 (Fig. 4B, Fig. 5A). In contrast, cultured pig LSEC efficiently phagocytosed resting baboon platelets, with a phagocytosis index of 258±27 (*P*<0.01) (Fig. 4B, Fig. 5A).

### Platelet phagocytosis by xenogeneic porcine LSEC is inhibited by blockade of the MAC-1 receptor

In order to elucidate pathways that may be involved in baboon platelet phagocytosis, we examined the effect of blocking known platelet receptors on the ability of porcine LSEC to phagocytose baboon platelets. Pre-incubation of cultured LSEC with ATA (blocks von Willebrand's factor; vWF), eptifibatide (blocks GpIIb/IIIa) or ICRF44 (anti-MAC-1 antibody) significantly prevented phagocytosis of baboon platelets, with ICRF44 being the most effective (*P*<0.01; Fig. 5B).

## Discussion

Elucidating molecular mechanisms of platelet consumption following liver xenotransplantation might lead to the development of new therapeutic strategies to prolong graft and recipient survival, as well as impact our basic understanding of platelet homeostasis. In this study, we showed that the formation of platelet aggregates and platelet phagocytosis by porcine LSEC previously detailed in *ex vivo* perfusion systems [9], also can be examined in a cell culture system. This model has allowed us to test for the involvement of multiple putative pathways involved in platelet activation and contrast their relative importance.

We confirmed that only xenogeneic hepatocytes, AEC, and LSEC induce aggregation of resting pig platelets. This observation could be explained by several inter-species differences already described. For example, porcine vWF constitutively induces human platelet aggregation via glycoprotein Ib, without the usual requirement for platelet activation or endothelial cell injury [14]. However, the degree of aggregation we noted in this current system was quite mild, and there were no differences noted between AEC and LSEC or hepatocytes. Thus, xenogeneic cell induced platelet aggregation is probably not responsible for the severe drop in platelet counts that is peculiar to liver xenotransplantation as compared to kidney or heart xenotransplantation. As previously noted, multiple anti-platelet agents tested are able to prevent xenogeneic cell aggregation responses.

However, porcine LSEC were uniquely active in phagocytosing resting baboon platelets, when compared to porcine AEC. This observation correlates with known properties of hepatic sinusoidal endothelium and the observation that profound thrombocytopenia and fatal hemorrhage is preferentially noted after liver xenotransplantation. Among the recognized mechanisms involved in platelet phagocytosis by macrophages is the recognition of clustered β-N-acetylglucosamine (beta GlcNAc) residues on same-species, or on chilled platelets by MAC-1 (α_M_β_2_ integrin, CD11b/CD18, CR3) on macrophages [15], [16]. Interestingly, blocking the beta-GlcNAc residues of GPIb with galactose prevents the binding of refrigerated human and mouse platelets by macrophages, prolonging the circulation time [17]. In addition, MAC-1 binds fibrinogen, which in turn, binds platelet receptor glycoprotein IIb/IIIa, representing a second pathway by which MAC-1 captures platelets for cellular interactions [18].

As we could identify the presence of MAC-1 on a subgroup of LSEC by flow cytometry, we tested several pharmacologic agents targeting MAC-1 recognition of platelets. Since beta-GlcNAc residues on GPIb are important in MAC-1 mediated platelet phagocytosis, and constitutive binding of porcine vWF to primate GPIb induces clustering of GPIb, we added ATA, a vWF inhibitor, to our system. ATA decreased xenogeneic platelet phagocytosis by approximately 80%. Next, we blocked the secondary recognition pathway with eptifibatide (EFT), a synthetic GP IIb/IIIa receptor antagonist currently used in the treatment of acute myocardial infarction and post-angioplasty stenosis [19]. It was slightly less effective than ATA in preventing platelet internalization. Although an anti-MAC-1 antibody almost completely abrogated porcine LSEC phagocytosis of baboon platelets, MAC-1 positive cells were only 14% of isolated LSEC. These observations suggest that only a sub-population of cells (MAC-1 positive LSEC only) is involved in xenogeneic platelet phagocytosis.

Our results demonstrate that glycoprotein - α_M_β_2_ integrin pathways are involved in the consumption of baboon platelets by porcine LSEC. It is likely that other receptor pathways are involved in the clearance of primate platelets after pig to baboon liver xenotransplantation, such as the Ashwell-Morell or asialoglycoprotein receptor pathway. This has been previously reported as a major signal for platelet homeostasis [20], in the xenotransplant setting [21] and was not a focus of the current work.

In summary, pig aortic endothelial cells, hepatocytes and liver endothelial cells all induce aggregation of baboon platelets to a similar, albeit mild extent, but only pig liver endothelial cells efficiently phagocytose baboon platelets. Blocking glycoprotein-αMβ2 pathway recognition pathways prevents internalization of baboon platelets by porcine LSEC, and could be used in a targeted manner to avoid the lethal complication of thrombocytopenia following porcine liver xenotransplantation into primates.
